# Efficacy and safety of voriconazole in the treatment of invasive pulmonary aspergillosis in patients with liver failure: study protocol for a randomized controlled clinical trial

**DOI:** 10.1186/s13063-023-07755-6

**Published:** 2023-12-17

**Authors:** Xue Yu, Lejia Xu, Jiaxing Zheng, Ziying Lei, Yihua Pang, Xiaojie Li, Jianyun Zhu, Jing Liu

**Affiliations:** 1 Department of Infectious Diseases, Third Affiliated Hospital of Sun Yat-Sen University, 600# Tianhe RoadGuangdong Province, Guangzhou City, 510630 China; 2 Department of Pharmacy, Third Affiliated Hospital of Sun Yat-Sen University, 600# Tianhe RoadGuangdong Province, Guangzhou City, 510630 China; 3 Department of Laboratory Medicine, Third Affiliated Hospital of Sun Yat-Sen University, 600# Tianhe RoadGuangdong Province, Guangzhou City, 510630 China

**Keywords:** Voriconazole, Invasive pulmonary aspergillosis, Acute-on-chronic liver failure, Study protocol

## Abstract

**Background:**

Acute-on-chronic liver failure (ACLF) is a common clinical type of liver failure, and patients with acute-on-chronic liver failure are prone to fungal infections, especially the increasing incidence of invasive pulmonary aspergillosis (IPA). Voriconazole is recommended as the first-line antifungal agent in the treatment of invasive aspergillosis; however, no recommendation has been given for patients with severe liver cirrhosis (Child–Pugh C) and liver failure. This trial aims to examine the therapeutic effects and safety of voriconazole in the treatment of IPA in patients with liver failure.

**Methods:**

This study is a non-double-blind randomized controlled trial. The 96 eligible acute-on-chronic liver failure patients complicated with invasive pulmonary aspergillosis will be randomly assigned to receive either the optimized voriconazole regimen or the recommended voriconazole regimen for patients with mild to moderate liver cirrhosis (Child–Pugh A and B), at a 1:1 ratio, with an 8-week follow-up period. The antifungal efficacy of voriconazole will be the primary outcome measure. Plasma voriconazole trough concentration, the laboratory examination (CRP, PCT, ESR, etc.), chest CT, adverse events, and mortality at week 4 and 8 will be the secondary outcome measures.

**Discussion:**

This trial aims to demonstrate the efficacy and safety of voriconazole in the treatment of IPA in patients with liver failure, which is expected to provide a reference for scientific optimization of voriconazole regimens and a realistic basis for the standardized treatment of acute-on-chronic liver failure patients complicated with invasive pulmonary aspergillosis.

**Trial registration:**

The trial was registered with the Chinese Clinical Trial Registry, ChiCTR2100048259. Registered on 5 July 2021.

## Administrative information

**Table:** 

Title {1}	Efficacy and safety of voriconazole in the treatment of invasive pulmonary aspergillosis in patients with liver failure: study protocol for a randomized controlled clinical trial
Trial registration {2a and 2b}	The trial was registered with the Chinese Clinical Trial Registry, ChiCTR2100048259. Registered on 5 July 2021
Protocol version {3}	Date:2021.11.30 Version identifier: 1.0
Funding {4}	This study was supported by China International Medical Foundation (grant number: Z-2018–35-2003) and Clinical Research Foundation of the 3rd Affiliated Hospital of Sun Yat-sen University(grant number: YHJH201904)
Author details {5a}	Xue Yu^1,#^, Lejia Xu^2,#^, Jiaxing Zheng^1^, Ziying Lei^1^, Yihua Pang^1^, Xiaojie Li^3^, Jianyun Zhu^1,*^, Jing Liu^1,*^ Department of Infectious Diseases, Third Affiliated Hospital of Sun Yat-Sen University, 600# Tianhe Road, Guangzhou city, Guangdong province, China, 510630 Department of Pharmacy, Third Affiliated Hospital of Sun Yat-Sen University, 600# Tianhe Road, Guangzhou city, Guangdong province, China, 510630 Department of Laboratory Medicine, Third Affiliated Hospital of Sun Yat-Sen University, 600# Tianhe Road, Guangzhou city, Guangdong province, China, 510630 #Xue Yu and Lejia Xu shared the first authorship *Co-corresponding authors
Name and contact information for the trial sponsor {5b}	China International Medical Foundation 42 Dongsi Xidajie, Beijing, 100710 Tel:86–10-85158267 Fax:86–10-65266642
Role of sponsor {5c}	The funder was not involved in trial design, conduct, or reporting All authors (Xue Yu, Lejia Xu, Jiaxing Zheng, Ziying Lei, Yihua Pang, Xiaojie Li, Jianyun Zhu, Jing Liu) were involved in the conceptualization and design of the trial, made significant intellectual contributions to the written protocol, and have approved the submitted version

## Introduction

### Background and rationale {6a}

Acute-on-chronic liver failure (ACLF) is a prevalent kind of liver failure in which patients acquire acute or subacute liver failure as a result of chronic liver disease [1]. Meanwhile, patients with acute-on-chronic liver failure are more susceptible to fungal infections, particularly the rising incidence of invasive pulmonary aspergillosis (IPA), which has been reported to be as high as 5.0–8.3% [2–4], with a significant increase in short-term mortality (73.5–100%) [2, 3, 5].

At present, amphotericin B, voriconazole, and echinocandins are three frequently prescribed drugs for IPA patients. Voriconazole is recommended as the first-line antifungal agent in the treatment of invasive aspergillosis in the guidelines of the Infectious Diseases Society of America in 2016 [6]. The routine intravenous dosing regimen for voriconazole is as follows: 6 mg per kilogram intravenously twice daily on day 1, then 4 mg per kilogram intravenously twice daily for at least 7 days, after which the patient may switch to oral voriconazole at 200 mg twice daily. The loading dose can make plasma trough concentration (*C*_min_) reach the steady-state concentration of voriconazole in the shortest time (24 h), and the final half-life of voriconazole is only 6 h, so it needs to be administered twice a day [7]. Its therapeutic effect and adverse reactions (such as hepatotoxicity, hallucination, mental abnormality, etc.) are closely related to plasma voriconazole trough concentration. Since voriconazole is mainly metabolized by the liver drug enzyme CYP450, liver injury caused by liver cirrhosis and liver failure may decrease the activity or number of metabolic enzymes, resulting in a decrease in voriconazole metabolism and a corresponding increase in the incidence of adverse reactions [7–10]. Voriconazole drug instructions indicate that for patients with mild to moderate liver cirrhosis (Child–Pugh A and B), the loading dose remains unchanged and the maintenance dose is halved. However, there is no recommendation for patients with severe liver cirrhosis (Child–Pugh C) and liver failure.

So far, there are few studies on the use of voriconazole in ACLF patients, and some scholars have analyzed its individualized scheme, but its safety is still unknown. We have retrospectively observed hospitalized ACLF patients complicated with IPA who were treated with voriconazole in our hospital. All of them received the recommended loading dose(400 mg intravenously twice daily on day 1) for Child–Pugh A and B cirrhosis patients, and the maintenance dose was based on the liver damage and their weight. We found that all the four cases’ plasma concentrations exceeded the safe range and then had hallucinations, mental symptoms, and liver toxicity. It can be found that for IPA, the recommended therapy for patients with mild to moderate liver cirrhosis is not suitable for ACLF patients. In addition, due to the slower metabolism in patients with liver failure, the half-life of voriconazole is prolonged and the steady-state time may be different from that of the normal population or patients with mild to moderate liver damage [11]. As a result, not only is it critical to find a more safe and effective administration regimen, but it is also necessary to monitor the plasma concentrations earlier and more closely to determine the best time for plasma concentration monitoring as well as to use voriconazole scientifically to avoid losing the opportunity to use the first-line antifungal agent in the therapy.

### Objectives {7}

Based on the pharmacokinetic profile of voriconazole, the objective of this trial is to examine the efficacy and safety of voriconazole in the treatment of IPA in patients with liver failure, to provide a reference for scientific optimization of voriconazole regimens and a realistic basis for the standardized treatment of patients with ACLF complicated with IPA.

### Trial design {8}

**Fig. 1:**
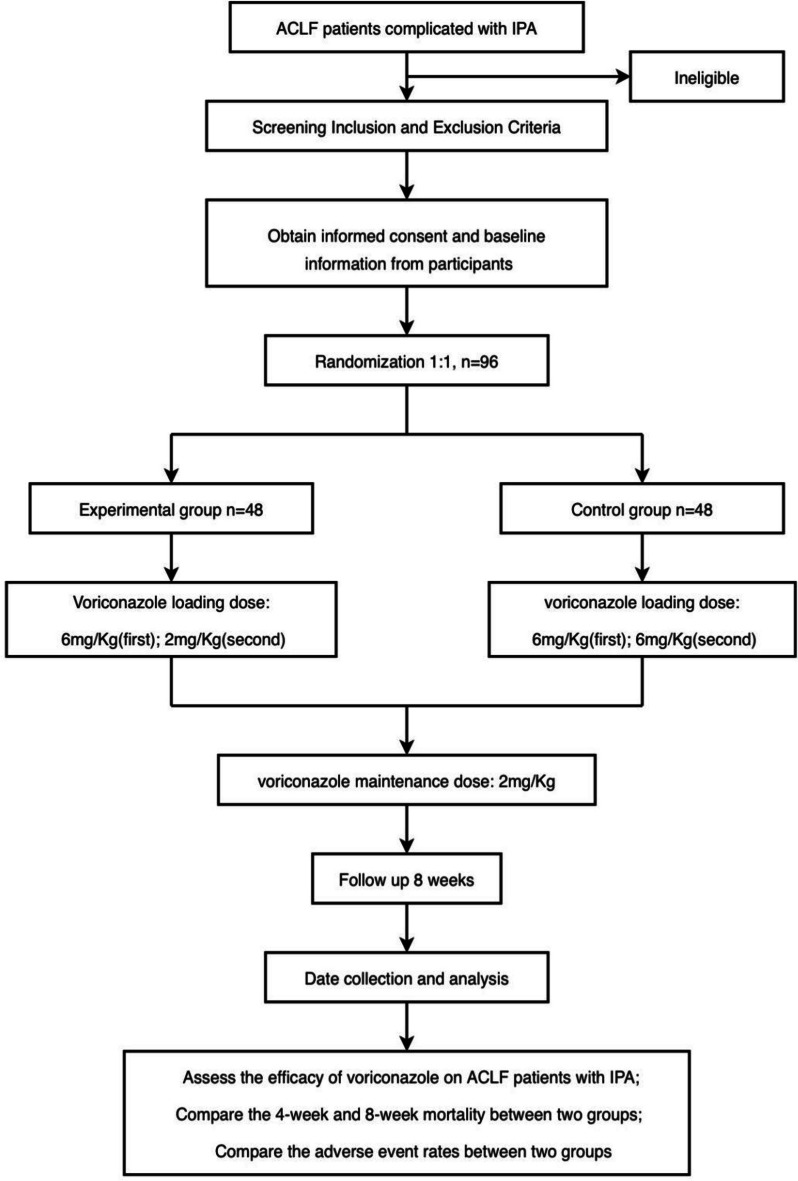
Flow chart of this study

This study is a non-double-blind, randomized controlled, parallel group and non-inferiority trial. A flowchart of the trial is shown in Fig. 1. Participants who meet the eligibility criteria will be randomized respectively into two groups as follows: (1) the experimental group and (2) the control group; they will receive different voriconazole regimens. The protocol reporting follows the Standard Protocol Items for Clinical Trials 2013 (SPIRIT 2013) [12].

## Methods: participants, interventions, and outcomes

### Study setting {9}

The study will be conducted in the Department of Infectious Diseases, The Third Affiliated Hospital of Sun Yat-Sen University, a large Grade-A Tertiary Hospital in Guangzhou.

### Eligibility criteria {10}

#### Inclusion criteria

The following are the inclusion criteria:

1. Patients 18–85 years of age
2. Patients with the signing of informed consent and confirming enrollment
3. ACLF was diagnosed according to guidelines for diagnosis and treatment of liver failure (2018) [13]: a presentation of extreme fatigue and severe gastrointestinal symptoms (anorexia, abdominal distension, nausea, and vomiting), progressive jaundice over a short period (total serum bilirubin ≥ 10 × ULN or a daily rise ≥ 17 μmol/L), a pronounced bleeding tendency (PTA ≤ 40% or INR ≥ 1.5), and any degree of potential complications (encephalopathy, clinical ascites, etc.) based on ongoing chronic liver diseases
4. IPA was diagnosed according to the latest EORTC/MGC consensus definition updated in 2020 [14, 15], including proven and probable IPA
  1. Proven IPA: histopathologic examination of a pulmonary specimen obtained by needle aspiration or biopsy in which hyphae or melanized yeast-like forms are seen accompanied by evidence of associated tissue damage or recovery of the aspergillus by the culture of the alveolar lavage fluid obtained by a sterile fiberoptic bronchoscope lavage from a normally sterile and clinically or radiologically abnormal site consistent with an infectious disease process
  2. Probable IPA: host factors + clinical features (fever, cough, sputum, and other symptoms; signs such as rales in the lungs; pulmonary imaging in support of aspergillus infection) + mycological evidence

#### Exclusion criteria

The following are the exclusion criteria:

1. Pregnant or lactating women
2. Patients with acute fatty liver in pregnancy
3. Patients with HIV co-infection
4. Patients accompany with severe underlying diseases
5. History of allergic to voriconazole

#### Dropout criteria

The following are the dropout criteria:

1. Patients with ALT or TBiL continuous elevation (more than 5 or 2 times higher than the baseline)
2. Patients with central nervous system toxicity such as hallucinations and psychiatric abnormalities and plasma voriconazole trough concentration persistently exceeding 6 mg/L, which cannot be relieved by reducing the dose to 50 mg qd
3. Patients who withdraw their consent during the study

### Who will take informed consent? {26a}

Informed consent will be obtained by the investigators.

There will be an investigator on our team who will be specifically responsible for engaging with patients to participate in the study. The investigator will be responsible for providing each patient and family member with a Participant Information and Consent Form (PICF) describing the purpose and procedures of the study, foreseeable benefits and potential risks of participation, information on data protection procedures, and the option to withdraw from the study at any time, and no reason is required, which should be read by the patient. The investigator will answer any questions the patient and family members may have, and both the patient or his or her family and the investigator will sign the informed consent form to demonstrate the patient’s and family’s full understanding of the study. Written informed consent must be obtained from all participants prior to any trial-related procedures and prior confirmation that inclusion/exclusion criteria are met during the eligibility assessment is required before patient enrollment.

### Additional consent provisions for collection and use of participant data and biological specimens {26b}

In the PICF, the participants will be informed about the use and storage of personal data and biological specimens collected during their participation in the trial. The PICF also contains information concerning the personnel who can access the data collected during this trial and the period that the data will be kept following the study’s completion. By signing the informed consent form, the participants agree to the terms addressed in the PICF.

## Interventions

### Explanation for the choice of comparators {6b}

Instruction of voriconazole shows no recommendation for patients with severe liver cirrhosis (Child–Pugh C) and liver failure, so the choice of comparators in this study is based on the recent research on related topics and the previous retrospective studies of our research group [3, 7, 10, 11, 16–18]. The recommended protocol of voriconazole for Child–Pugh A or B liver cirrhosis was chosen as a control to best determine any effects of the interventions.

### Intervention description {11a}

Eligible patients will be randomized to either the experimental group or the control group. According to the recent research on related topics and the previous retrospective studies of our research group, the experimental group will receive the optimized voriconazole regimen: first loading dose, 6 mg per kilogram intravenously; second loading dose (twelve hours interval from first dose), 2 mg per kilogram intravenously; maintenance dose, 2 mg per kilogram intravenously twice daily.

### Criteria for discontinuing or modifying allocated interventions {11b}

Persistent high trough concentrations are an independent risk factor for hepatic and central toxicity of voriconazole. Therefore, if ALT or TBiL is persistently elevated (i.e., a fivefold increase for ALT or a twofold increase for TBil above baseline) or central nervous system toxicity such as hallucinations and psychiatric abnormalities occur, and plasma voriconazole trough concentration persistently exceeding 6 mg/L, which cannot be relieved by reducing the dose to 50 mg qd, voriconazole can be discontinued and replaced with echinocandins for antifungal treatment or even withdrawn from the trial. A patient may also be withdrawn from the study for the following reasons: (1) lost to follow-up or (2) withdrawal of consent.

### Strategies to improve adherence to interventions {11c}

The following measures will be, and or have been, taken to improve adherence to interventions and follow-up:

1. All participants will be informed of the study procedures as well as potential benefits and risks to make them fully understand the significance of their involvement in the study.
2. All participants will receive two free voriconazole blood tests and will be able to use first-line therapeutic agents with closely monitored drug blood levels and adverse effects for better outcomes.

### Relevant concomitant care permitted or prohibited during the trial {11d}

There will be no restrictions regarding concomitant care during the trial.

### Provisions for post-trial care {30}

All patients will return to standard care after the trial.

### Outcomes {12}

#### Primary outcome

The primary endpoint will be the antifungal efficacy of voriconazole. All patients treated with voriconazole were systematically evaluated by the same investigator on the team. Each patient was evaluated on the basis of case report, imaging (computed tomography scan, CT) findings. CT images of the lung were reviewed by two radiologists. Changes in lesions were assessed visually using computed planimetry to help estimate percentage change. The response categories for complete response, partial response, and treatment failure were the same as those in similar previous studies [16–19]. Complete responses were defined by the resolution of all clinical signs and symptoms and more than 90% of the lesions due to invasive aspergillosis that were visible on radiology. Partial responses were defined by clinical improvement and greater than 50% improvement in findings on radiology. Stable responses were defined by the absence of change from base line or an improvement of less than 50%. Failure of therapy encompassed progression and death due to IPA. Complete and partial responses were classified as successful outcomes.

#### Secondary outcomes

The secondary endpoints will include the following. Half an hour before drug administration, voriconazole plasma concentrations, and laboratory tests of participant were recorded; laboratory tests including C-reactive protein (CRP), procalcitonin (PCT), and erythrocyte sedimentation rate (ESR), etc. Meanwhile, adverse events and mortality at weeks 4 and 8 will also be recorded.

### Participant timeline {13}

The participant timeline is presented in Table 1.

**Table 1:** The participant timeline

Viewpoint	V1	V2	V3	V4	V5	V6	V7	V8	V9	V10	V11
Time point	0 (baseline)	12 h	24 h	48 h	72 h	Week 1	Week 2	Week 3	Week 4	Week 6	Week 8
Use of voriconazole	Before treatment	Before the 2nd dose	Before the 3rd dose	Before the 5th dose	Before the 7th dose	Week 1	Week 2	Week 3	Week 4	Week 6	Week 8
Time-window	− 7	0	0	0	0	± 1	± 2	± 2	± 2	± 3	± 3
Informed consent	√										
Essential information	√										
Inclusion/exclusion criteria	√										
Previous treatments	√										
Previous medicine	√										
CYP2C19 gene	√										
Symptoms	√	√	√	√	√	√	√	√	√	√	√
Physical sign	√	√	√	√	√	√	√	√	√	√	√
Comorbidity	√	√	√	√	√	√	√	√	√	√	√
ECG	√										
Lung CT	√						√		√	√	√
Abdominal ultrasonography	√								√		√
Blood routine	√				√	√	√	√	√	√	√
Renal function	√								√		√
Inflammation indicators	√				√	√	√	√	√	√	√
G/GM test	√					√	√	√	√	√	√
BALF culture or histopathology	√										
Biochemical test	√				√	√	√	√	√	√	√
Coagulation function	√				√	√	√	√	√	√	√
ABG	√				√	√	√	√	√	√	√
Blood ammonia	√				√	√	√	√	√	√	√
Pregnancy test (female)	√										
Plasma voriconazole trough concentration		√	√	√	√	√	√	√	√	√	√
Concomitant medication	√	√	√	√	√	√	√	√	√	√	√
Child–Pugh classification	√					√	√	√	√	√	√
MELD score	√					√	√	√	√	√	√
Condition evaluation		√	√	√	√	√	√	√	√	√	√
AE	√	√	√	√	√	√	√	√	√	√	√
SAE		√	√	√	√	√	√	√	√	√	√

### Sample size {14}

We referred to a study about drug monitoring and safety of voriconazole therapy in patients with Child–Pugh B and C cirrhosis; this study suggested that about 30% of patients in group B (loading dose, 200 mg intravenously twice daily on day 1; maintenance dose: 100 mg intravenously twice daily) had voriconazole *C*_min_ > 5 mg/L on the third day of treatment [20].

Based on pharmacokinetic knowledge, we estimate that the rate of patients in the experimental group in this study with *C*_min_ > 5 mg/L after 3 days of treatment with voriconazole will not be lower than that in group B described above. The resulting sample size was calculated as follows:

$${N}_{c}={\left({Z}_{1-\alpha /2}\sqrt{\overline{P }\left(1-\overline{P }\right)\left(1+r\right)/r} +{Z}_{1-\upbeta }\sqrt{{P}_{c}\left(1-{P}_{c}\right)+{P}_{T}\left(1-{P}_{\mathrm{T}}\right)/r}\right)}^{2}/{\left({P}_{c}-{P}_{T}\right)}^{2}$$

NC: control group sample size.

PC (control group rate): 0.6, PT (experimental group rate): 0.3,

$\overline{P }$ (mean of control group rate and experimental group rate): 0.45.

*r*: grouping ratio, *r* = 1 for 1:1 grouping.

*α*: type I error; *β*: type II error.

Here $$\alpha =0.05, \beta =0.1, Z1-\frac{\alpha }{2}=1.96, Z1-\beta =1.28$$

Substituting into the formula: NC ≈ 42.16. By grouping 1:1, then at least 43 cases were taken from each group. Considering 10% dropout, the study will require recruiting approximately 96 participants for all the groups (*n* = 48 in each group) to adequately test the assumption.

### Recruitment {15}

The subjects will be recruited from the inpatients in the Infectious Department of the Third Affiliated Hospital of Sun Yat-Sen University. Recruitment is expected to begin in August 2021 and end in December 2023.

## Assignment of interventions: allocation

### Sequence generation {16a}

After inclusion in the trial, the randomization adopts a simple randomization method, and random numbers are generated through computer software (SAS9.4) by the statistician.

### Concealment mechanism {16b}

The method of allocation concealment is envelope concealment. Random numbers will be put into uniform envelopes. Then, subjects will be given uniform envelopes according to the order of inclusion and will be assigned random numbers in the envelopes.

### Implementation {16c}

After the participant’s eligibility has been confirmed and informed consent has been received, the participant will be randomized into the trial. A form will be provided to investigators and will be used to collate the necessary information prior to randomization. Only when all eligibility criteria and baseline data items have been provided a trial number will be allocated. Participants will be randomized at the level of the individual in a 1:1 ratio to either the experimental group or the control group. The interventions will be implemented based on randomization by investigators.

## Assignment of interventions: blinding

### Who will be blinded {17a}

This study is a non-double-blind trial.

### Procedure for unblinding if needed {17b}

The design is open label, so unblinding will not occur.

## Data collection and management

### Plans for assessment and collection of outcomes {18a}

A predeveloped questionnaire will be completed with patients’ socio-demographic characteristics and required baseline clinical information.

The rest of the information required for the experiment will be collected and assessed as follows:

1. Evaluation of plasma voriconazole trough concentration measurement: the measurement will be carried out half an hour before the second, third, fifth, seventh, and fifteenth doses, once a week after the fifteenth dose, and every 2 weeks after week 4
2. Evaluation of laboratory examination (including the following: blood routine test, CRP, PCT, ESR, FER, LDH, 1,3-β-d-Glucan test (G test), galactomannan test (GM test), hepatic and renal function, coagulation function, arterial blood gas analysis, and blood ammonia): weeks 1, 2, 3, 4, 6, and 8 after the start of the study
3. Evaluation of imaging tests (including chest CT): once every 2 weeks
4. Evaluation of Child–Pugh score and model for end-stage liver disease (MELD) scores: weeks 1, 2, 3, 4, 6, and 8 after the start of the study

Schedule of assessments is detailed in Table 1.

### Plans to promote participant retention and complete follow-up {18b}

The purpose and importance of the trial will be explained through the research team and PICF during participant recruitment. Participants have the right to withdraw from the trial at any time and for any reason without compromising their medical care and routine treatment by the treatment team or institution. If a participant withdraws from the trial before the end of the study and because the data will be recorded anonymously, an intention-to-treat analysis will be performed on the data for such patients. If a patient withdraws consent during the conduct of the trial and does not wish to consent to the use of data already collected, and if these particular data are retrievable, the investigator will delete the data accordingly.

### Data management {19}

Data will be collected through a paper-based questionnaire, and the collected information will remain anonymous. All data recorded on paper forms will be safely stored in the Department of Infectious Diseases, The Third Affiliated Hospital of Sun Yat-Sen University, Guangzhou, China, following data protection procedures. Data will be entered using a fixed dual researcher and manually reviewed to avoid data entry errors. Data will be saved in Microsoft Excel spreadsheets and stored on the Department of Infection data system of the Third Affiliated Hospital of Sun Yat-sen University.

### Confidentiality {27}

Participants will be assigned a subject’s number for de-identification purposes and all the collected data tables will be identified by the subject’s number only. All data in this study will be stored in a special filing cabinet in the Third Affiliated Hospital of Sun Yat-Sen University. At the same time, we will set access to the file to ensure the security and confidentiality of the data. Only researchers from the Third Affiliated Hospital of Sun Yat-Sen University who participate in this research will have the authorization to access these stored documents.

### Plans for collection, laboratory evaluation, and storage of biological specimens for genetic or molecular analysis in this trial/future use {33}

The collected samples will be sent to the Laboratory of the Third Affiliated Hospital of Sun Yat-sen University for analysis in the first instance, after which they will be discarded instead of being preserved.

## Statistical methods

### Statistical methods for primary and secondary outcomes {20a}

In this study, a separate statistical analysis plan will be developed to analyze the data of the treatment group and the control group. The analyses will be based on Consolidated Standards of Reporting Trials guidelines, and all analyses will be on the principle of intention-to-treat, that is, all participants will be analyzed even if some subjects will fail to follow the original plan during the study. This analysis method can better evaluate the effect of the intervention, reduce bias, and be closer to the actual clinical situation. Our researchers are responsible for the follow-up to ensure that relatively complete data are obtained. Subjects who lacked data on the main results will be excluded in the study from the beginning, including missing CYP2C19 gene, lung CT, abdominal ultrasound, G/GM test, Child–Pugh score, and MELD score.

The PASW18.0 statistical software will be used to analyze the data. The counting data will be expressed by rate, and the rate will be compared by the chi-square test. Continuous normal distribution data will be expressed as mean ± standard deviation, *t*-test will be used for comparison between the two groups, non-normal data will be expressed as median (quartile spacing), Mann–Whitney test method will be used for comparison between the two groups, and *χ*^2^ test will be used for counting data. *P* < 0.05 indicates that the difference is statistically significant. The GraphPadPrism5 software will be to draw.

### Interim analyses {21b}

No interim analyses are planned.

### Methods for additional analyses (e.g., subgroup analyses) {20b}

No additional analyses are planned.

### Methods in analysis to handle protocol non-adherence and any statistical methods to handle missing data {20c}

No imputation of missing data will be performed for statistical analysis.

### Plans to give access to the full protocol, participant-level data, and statistical code {31c}

The complete trial protocol will be shared upon reasonable request. Group-level anonymized data may be shared with external investigators at the end of the trial after obtaining institutional approval to release the data externally. The results of the trial will be published in peer-reviewed journals and presented at conferences. Study results will be released to participating patients.

## Oversight and monitoring

### Composition of the coordinating center and trial steering committee {5d}

The trial will be conducted by the study research team, including the Infectious Diseases Department staff and masters. The principal investigator takes responsibility for the supervision of the trial and ensures compliance with the study protocol. The statistical research plan and statistical analysis will be supervised by the Research and Ethics Unit, The Third Affiliated Hospital of Sun Yat-Sen University.

### Composition of the data monitoring committee, its role and reporting structure {21a}

The data monitoring committee is unnecessary in this trial because this study will not involve participants with severe diseases and communication disabilities or interventions that can risk participants’ lives. Instead, the oversight of data quality will be provided by the Research and Ethics Unit, The Third Affiliated Hospital of Sun Yat-Sen University.

### Adverse event reporting and harms {22}

All participants will be monitored concerning any possible adverse events related to the administration of interventions, such as exacerbation of IPA, deterioration of liver function, and the emergence of other infections. As such, all adverse events (AEs) observed or reported by the patient are collected and evaluated for relatedness to trial intervention, seriousness, severity, expectedness, and outcome. AEs are defined in the Good Clinical Practice (GCP) guideline.

### Frequency and plans for auditing trial conduct {23}

The study team will audit the data regularly. The audit may be performed by The Third Affiliated Hospital of Sun Yat-Sen University Research and Ethics Unit’s staff to evaluate the clinical study conduct and compliance with the protocol, standard operating procedures, GCP, and the applicable regulatory requirements.

### Plans for communicating important protocol amendments to relevant parties (e.g., trial participants, ethical committees) {25}

The principal investigator will be responsible for any protocol amendments and their follow-up process. Protocol amendments will be submitted to the ethics committee and implemented upon approval. The principal investigator is responsible for disseminating changes to the protocol, and all study team members will receive adequate training on protocol amendments.

### Dissemination plans {31a}

The final data will be publicly disseminated. The results will be presented at relevant meetings and published in appropriate journals after the trial and analysis.

## Discussion

Although voriconazole is recommended as the first-line antifungal agent in the treatment of IPA in the guidelines of the Infectious Diseases Society of America in 2016 [6], there is still a lack of recommended dosing regimens or dosing consensus for ACLF patients complicated with IPA. A recent study on the pharmacokinetic changes of voriconazole in a population of patients with Child–Pugh class C cirrhosis or liver failure found no significant difference in the apparent volume of distribution (Vd) of voriconazole, while the clearance (CL) decreased significantly [20], which is consistent with the pharmacokinetic properties of voriconazole. The use of voriconazole in ACLF patients has also been evaluated in a retrospective analysis, where a dosing regimen with both loading and maintenance doses halved in the experimental group revealed that 30% of steady-state plasma voriconazole trough concentration still exceeded 5 mg/L after 3 days of dosing [20]; moreover, the time to reach steady-state was prolonged due to the lower loading dose, and the timing of plasma voriconazole trough concentration that could be monitored to guide dose adjustment was delayed accordingly [13]. According to the pharmacokinetic study of voriconazole, it is possible that the dose of 3 mg/kg may not reach the required plasma voriconazole trough concentration for most of the first day, which may delay the timing of treatment [21].

As a result, for the first time in a prospective randomized controlled trial, antifungal medication with varied loading doses of voriconazole will be delivered to ACLF patients complicated by IPA, and its efficacy and safety will be assessed by closely monitoring blood levels and adverse effects. The findings of this study may help close the gap in voriconazole dose recommendations for patients with liver failure, as well as revise guidelines for the diagnosis and treatment of people with liver failure complicated with IPA.

## Trial status

Protocol version number: 1

Protocol date: 30 November 2021.

Recruitment start date: August 2021.

Planned recruitment end date: December 2023.

## Data Availability

Any data required to support the protocol can be supplied on reasonable request.

## Abbreviations

ACLF: Acute-on-chronic liver failure
IPA: Invasive pulmonary aspergillosis
EORTC/MGC: The European Organization for Research and Treatment of Cancer and the Mycoses Study Group Education and Research Consortium
ALT: Alanine aminotransferase
TBiL: Total bilirubin
CRP: C-reaction protein
PCT: Procalcitonin
ESR: Erythrocyte sedimentation rate
FER: Ferritin
LDH: Lactate dehydrogenase
ECG: Electrocardiography
G test: 1,3-β-D-Glucan test
GM test: Galactomannan test
ABG: Blood gas analysis
MELD score: The model for end-stage liver. disease
AE: Adverse event
SAE: Severity adverse event
GCP: Good Clinical Practice
PICF: Participant information and consent form
WHO: World Health Organization
